# A Composite Renal Tumor with Dual Differentiation, Chromophobe and Collecting Duct Carcinoma

**DOI:** 10.1155/2018/2410920

**Published:** 2018-08-30

**Authors:** Lauren Pearson, Douglas J. Taatjes, Michele von Turkovich, Benjamin J. King, Maryam Zenali

**Affiliations:** ^1^Department of Pathology and Laboratory Medicine, University of Vermont Medical Center, USA; ^2^Microscopy Imaging Center, Larner College of Medicine, University of Vermont, USA; ^3^Department of Urology, University of Vermont Medical Center, USA

## Abstract

Chromophobe carcinoma constitutes a small subset of all renal carcinomas. Within this category, rare tumors with divergent differentiation have been recognized. Herein, we report a rare case of composite chromophobe and collecting duct carcinoma and describe its pathologic and clinical features.

## 1. Introduction

Subtypes of renal carcinoma include clear cell, papillary, chromophobe, medullary, and collecting duct carcinoma. Chromophobe carcinoma (ChC) comprises approximately 5% of all renal cell carcinomas (RCC) [1]. ChC is presumably derived from the cortical collecting ducts and is characterized by polygonal, clear to eosinophilic cells with distinct borders and often hyperchromatic, irregular (raisinoid) nuclei. Classically these cells have a perinuclear halo [2–4].

Chromophobe carcinoma is typically associated with a favorable prognosis, but aggressive behavior can be seen in a subset, including in hybrid tumors with a higher-­‐grade component. Transformation of ChC is a well-recognized phenomenon. Although uncommon, chromophobe carcinomas with divergent rhabdoid, osteosarcoma, liposarcoma, or spindle cells sarcoma-like differentiation are reported in the literature [5–12].

This paper describes another unusual composite renal tumor, predominantly ChC but with a minor component of collecting duct carcinoma (CDC). In parallel with ChC, collecting duct carcinoma is also uncommon and is believed to arise from the distal nephron, yet, in contrast to the majority of ChC, CDC pertains a poor prognosis.

## 2. Material and Method

The entire renal tumor was submitted for histologic examination. Formalin fixed paraffin embedded tissues were serially sectioned into 4 *μ*-thick sections and stained with Hematoxylin and Eosin (H&E), mucin, Hale colloidal iron, and a panel of immunoperoxidase stains.

Antibodies against CK7 (OV-TL, Thermo Scientific), vimentin (V9, Dako), c-kit (EP10, Leica), PAX-2 (polyclonal, Invitrogen), PAX-5 (SP34, Thermo Scientific), Racemase (P504, Ventana), and E-cadherin (NCH-38, Dako) were utilized. Immunostaining was performed using standard techniques by automated systems (Leica bond and Ventana systems), which includes a heat-induced epitope retrieval (HIER) at pH8.0 using EDTA. On both machines, a polymer is used to highlight the antigen-antibody reaction.

We performed electron microscopy (EM) on the blocks containing both tumor components. Briefly, the paraffin was removed with xylene from a 25 *μ*-thick section, followed by running the sample through a graded series of ethanols, and fixation in 2.5% glutaraldehyde/1% paraformaldehyde in 0.1 M cacodylate buffer for 30 minutes at 4°C. Following rinses in buffer, the sample was encased in 2% SeaPrep agarose, the agarose was crosslinked by placing in the same fixative as above, and areas of interest were cut from the whole sample and postfixed in 1% OsO_4_ in cacodylate buffer, rinsed, and stored in buffer at 4°C overnight. The small sample pieces were then dehydrated in a graded series of ethanols through propylene oxide and embedded in Spurr's resin. Following polymerization of the resin, 1 *μ*-thick sections were cut on an ultramicrotome with glass knives, stained with toluidine blue, and evaluated by light microscopy for regions of interest. Ultrathin sections were then cut with a diamond knife, retrieved onto 150-mesh nickel grids, contrasted with uranyl acetate and lead citrate, and examined with a JEOL JEM 1400 transmission electron microscope (JEOL USA, Inc., Peabody, MA) operating at 80 kV. Images are acquired in TIF format with an AMT XR611 ccd camera and are presented without processing.

## 3. Case Report

A 69-year-old woman with a history of hypertension was identified to have an incidental renal tumor on computed tomography (CT). She denied hematuria, lower urinary tract symptoms, pain, fever, fatigue, or weight loss. Her medical history was significant for hypertension and obstructive sleep apnea. Family history and social history were noncontributory. Physical exam at the time of presentation was normal.

On CT, the tumor was an exophytic, enhancing mass (3.0 × 2.0 × 3.5 cm), arising from the lower lateral pole of the left kidney with areas of low attenuation at its inferior aspect. The remainder of the urinary system was normal. No adenopathy or sign of metastasis was detected. An imaging obtained later the same year demonstrated no interval change in the size of the lesion. Biopsy was positive for an oncocytic neoplasm, which at the time was classified as an onocytoma. The patient was managed conservatively and presented 2 years later for repeat imaging.

A repeat CT was significant for an interval increase in the size of the mass from 3.5 to 5.6 cm in the greatest dimension. Tumor compressed the lower pole calyces without ureteral obstruction. There was no radiologic evidence of tumor calcification, fat, or infiltration into the adjacent tissues. Fine needle aspiration and the biopsies of the mass were again consistent with an oncocytic neoplasm.

**Figure 1:**
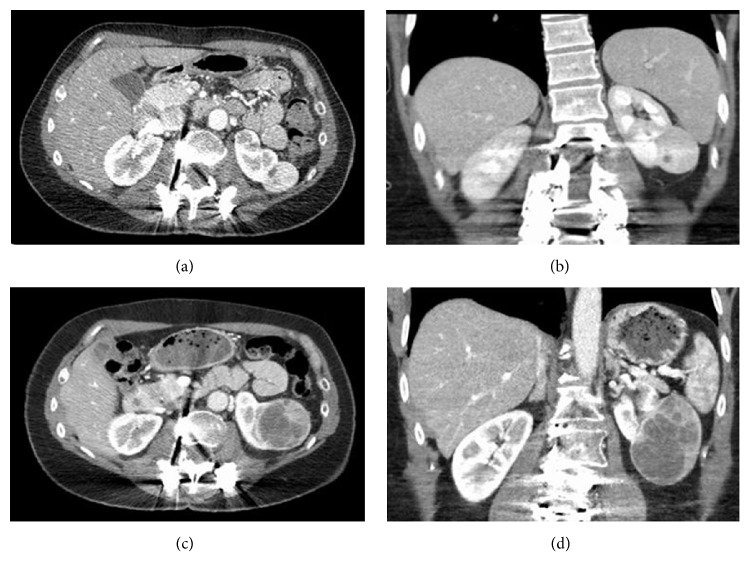
(a)–(d) CT axial and coronal scans depicting an exophytic mass in the lower pole of the left kidney: (a, b) initial presentation showing a well-circumscribed, enhancing mass with two small discrete, low attenuation areas in the inferior portion. (c, d) Enlargement of the renal tumor to almost twice the size from prior examination with compression of calyces, increased heterogeneity, and enhancement at the peripheral and anterior aspects of the mass.

Comparative radiologic images are provided (Figure 1); the top images are radiographs with a smaller tumor from 2 years ago, while the bottom radiographs are from the patient's recent CT with a larger tumor. Given the unusual clinical features and behavior of the tumor, the patient was referred for a radical nephrectomy.

**Figure 2:**
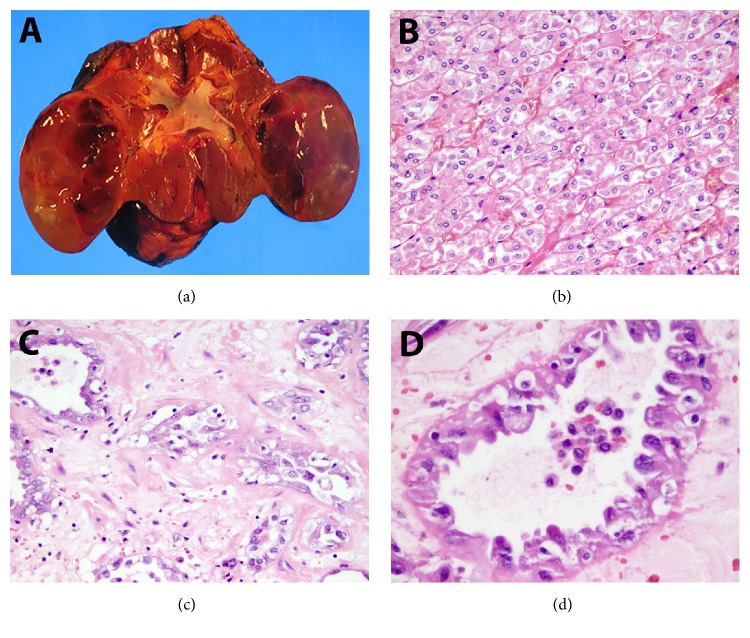
(a)-(d) Gross image and representative microscopic images: (a) Solid and cystic mass with a tan-brown cut surface involving the lower pole of the kidney. (b) Representative illustration from the dominant portion of the tumor, an eosinophilic variant of ChC containing relatively uniform cells with distinct borders and central nucleus. (c)-(d) A minor component of tumor with a tubulocystic morphology, marked cellular atypia, and hobnail nuclear features, in keeping with CDC, low, and high magnifications provided.

The specimen received at the pathology lab was composed of an intact kidney and perinephric adipose tissue (205g, 8.5 × 8.0 × 4.2 cm). At the midlower pole of the kidney, there was a circumscribed, cystic, and focally solid mass (6.0 × 4.7 × 4.5 cm). The mass had a tan-brown multiloculated cut surface (Figure 2(a)). There was no gross invasion of the renal vessels, ureter, or perinephric fat.

The majority of tumor was composed of monotonous cells with distinct borders, abundant eosinophilic cytoplasm, raisinoid nuclei and perinuclear halos, raising a possibility of an eosinophilic chromophobe carcinoma. As anticipated, this cellular component had cytoplasmic staining with Hale colloidal iron and membranous staining with c-kit (CD 117) and Ec-adherin. It was negative with vimentin.

**Figure 3:**
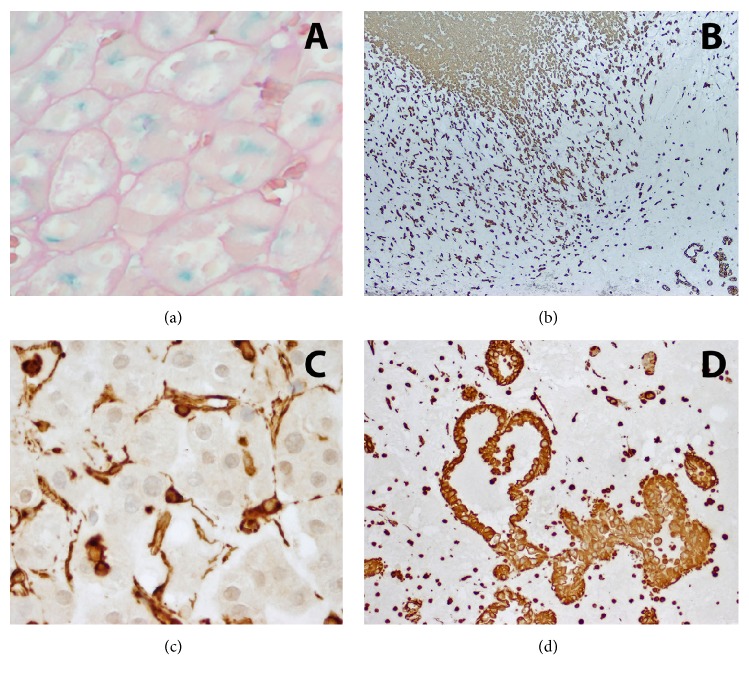
(a)-(d) Staining pattern of different tumor components: (a) positive intracellular staining with Hale colloidal iron in ChC. (b) CK7 stain highlighting both ChC and CDC components, at their emerging front. (c) While vimentin stain is negative in the ChC tumor cells, only highlighting vasculature, (d) it is diffusely positive in the CDC component.

A smaller subset of tumor cells had increased atypia, higher grade, hobnail morphology, and a tubulocystic architecture set within a desmoplastic stroma. This component was negative with Hale-Colloidal Iron and Racemase and had foci of intraglandular staining with mucin. CK7 and PAX-2 were positive but PAX-5 was negative in both components. Vimentin was only positive in the higher-grade tumor component, morphologically and by immunoprofile consistent with collecting duct carcinoma (see Figures 2 and 3 for the H&E and staining images).

Proliferative index, assessed by Ki-67 labeling, was low in the chromophobe (0–5% nuclear staining) and high in the collecting duct carcinoma component (60% of nuclei).

**Figure 4:**
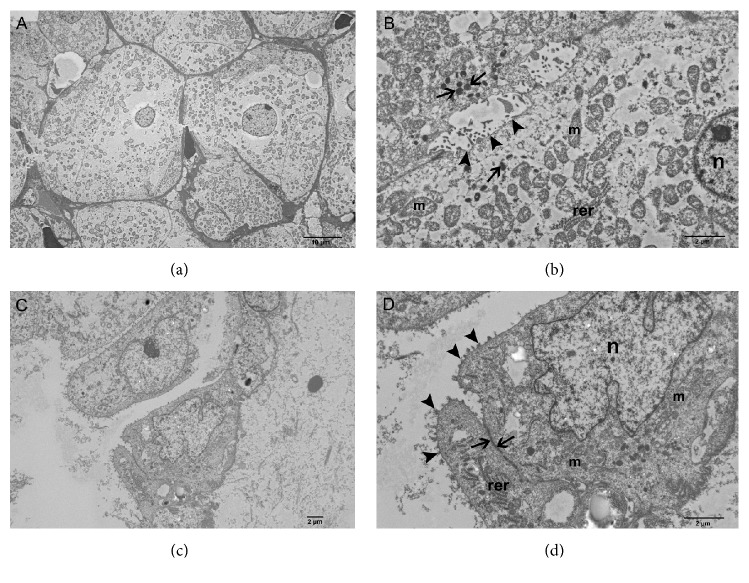
(a)-(d) Two different areas of tumor retrieved from the paraffin embedded sample and processed for EM: (a) Low magnification image depicting overall ultrastructural features of the peripheral tumor cells. These cells are round-ovoid and have a central nucleus, mitochondria and cytoplasmic microvesicles. (b) Higher magnification shows plasma membrane border separating two tumor cells (arrowheads), nucleus (n), abundant mitochondria (m), dense cytoplasmic vesicles (arrows), and patches of rough endoplasmic reticulum (rer). (c) Low magnification image of high grade tubulocystic tumor cells. The cells are epithelioid and have large, irregularly shaped nuclei. (d) Higher magnification shows cytoplasm with mitochondria (m), patches of rough endoplasmic reticulum (rer), irregularly shaped nucleus (n), microvillus studded apical plasma membrane (arrowheads), and junctional complex between the adjacent tumor cells (arrows).

On EM analysis, although the morphologic preservation was somewhat compromised by formalin fixation and paraffin embedding, ultrastructural details of the two types of tumor cells were readily visible and distinctive. The tumor section represented by monotonous cells contained rounded cells with centrally located nucleus (Figure 4(a)). At higher magnification, their cytoplasm was found to be packed with mitochondria and prevalent electron dense microvesicles (Figure 4(b)). The central tubulocystic-appearing regions contained elongated epithelioid cells with large, irregularly shaped nuclei (Figure 4(c)). At higher magnification, the plasma membrane of these cells possessed abundant microvillar projections with junctional complexes joining adjacent cells (Figure 4(d)). The cytoplasm was rich with organelles including mitochondria and rough endoplasmic reticulum.

## 4. Discussion

Classes of RCC are recognized based on their morphology, molecular, and immunophenotypic properties. ChC constitutes a small percentage of all RCC and when compared with the more prevalent clear cell carcinoma has a better prognosis [1]. Rare composite and/or dedifferentiated ChC with papillary and collecting duct component or rhabdoid, sarcomatous, or neuroendocrine features have been recognized [4–12]. Sarcomatous change is reported in up to 7.6% of all ChC and is more likely associated with a poor outcome [4, 6, 13].

Conventional ChC is composed of medium-sized polygonal cells with raisinoid nuclei, pale to eosinophilic cytoplasm, and prominent halo, arranged in solid and alveolar structures. Hale colloidal iron has diffuse cytoplasmic staining in chromophobe carcinoma. ChC is also positive with CK7 and CD 117 but is negative with CK20 and vimentin [7].

Although ChC is typically localized and has a favorable prognosis, aggressive behavior and metastatic lesions are observed; accuracy of morphologic typing and quantification of various tumor elements have significant clinical implications. Metastatic ChC is reported to have a higher incidence of sarcomatous and/or dedifferentiated component [4, 6].

Although most of renal epithelial tumors arise from the proximal nephron, a minority originate from the distal nephron. In our case, tumor had distinct areas of ChC and collecting duct carcinoma (CDC); as both these components are suspected to arise from the distal nephron, such divergent differentiation is not entirely unforeseen. Renal cancer with overlapping distal nephron morphology and karyotype has been reported [14].

CDC is a highly aggressive tumor with a poor prognosis (mean survival 11.5 months) that arises from the renal medulla. Its histogenesis is debatable, although a putative origin from the distal collecting ducts has been accepted. The usual histopathologic pattern of CDC is that of a tubulopapillary or tubulocystic carcinoma within a desmoplastic stroma that often contains neutrophils [14–17]. Bizarre glandular elements are lined by high-grade, cuboidal, or hobnail cells.

Although, in our case, initially the tumor was slow in growth, it had an unexpected accelerated growth during the clinical follow-up, perhaps in association with an evolving higher-grade component. The aforementioned emphasizes the importance of clinic-radiologic surveillance and adequate and generous sampling, as a divergent component may arise in association with ChC.

While ChC constituted 95% of the tumor, collecting duct carcinoma comprised only 5% of the total tumor volume and only involved the center of the mass. There was no lymph-vascular or perineural invasion. Considering very scant and confined nature of the CDC (localized to the center of the mass) and in parallel lack of lymph-vascular invasion, it would not be unexpected if this composite tumor is closer in biologic behavior to ChC than to CDC.

Thus, it is worth including in the diagnostic report not only the morphologic types but also the proportions, volume, and subsites of different neoplastic components. Of note, at five-year follow-up with radiographic surveillance, our patient has remained disease-free with no evidence of metastatic disease.
